# Methylation related genes affect sex differentiation in dioecious and gynodioecious papaya

**DOI:** 10.1093/hr/uhab065

**Published:** 2022-01-20

**Authors:** Ping Zhou, Xiaodan Zhang, Xinyi Ma, Jingjing Yue, Zhenyang Liao, Ray Ming

**Affiliations:** 1 Fruit Research Institute，Fujian Academy of Agricultural Sciences，Fuzhou 350013，Fujian, China; 2 Department of Plant Biology, University of Illinois at Urbana-Champaign, Urbana, IL 61801, USA; 3 FAFU and UIUC Joint Center for Genomics and Biotechnology, Fujian Provincial Key Laboratory of Haixia Applied Plant Systems Biology, Fujian Agriculture and Forestry University, Fuzhou 350002, Fujian, China

## Abstract

Morphological, genic and epigenetic differences often exist in separate sexes of dioecious and trioecious plants. However, the connections and relationships among them in different breeding systems are still unclear. Papaya has three sex types, which is genetically determined and epigenetically regulated, and was chosen as a model to study sex differentiation. Bisulfite sequencing of genomic DNA extracted from early-stage flowers revealed sex-specific genomic methylation landscapes and seasonally methylome reprogramming processes in dioecious and gynodioecious papaya grown in spring and summer. Extensive methylation of sex-determining region (SDR) was the distinguishing epigenetic characteristics of nascent XY sex chromosomes in papaya. Seasonal methylome reprogramming of early-stage flowers in both dioecy and gynodioecy systems were detected, resulting from transcriptional expression pattern alterations of methylation-modification-related and chromatin-remodeling-related genes, particularly from those genes involved in active demethylation. Genes involved in phytohormone signal transduction pathway in male flowers have played an important role in the formation of male-specific characteristics. These findings enhanced the understanding of the genetic and epigenetic contributions to sex differentiation and the complexity of sex chromosome evolution in trioecious plants.

## Introduction

Flowers develop from the floral meristem initiated in specific positions when endogenous developmental signals coordinate with external environmental cues to switch from vegetative to reproductive phase. For most cases, the formation of flowers has gone through evolutionarily conserved processes, including floral meristem specification, floral patterning, and floral organ specification as well as morphogenesis. During these processes, male (stamens) and female (carpels) sexual organs are initiated and then differentiated in the inner two whorls, which are surrounded by outer two whorls of perianth. Many regulatory pathways were demonstrated to control and involve in flower development, including transcriptional circuits and their interplay with phytohormones [1]. Together with inherently genetic regulation, epigenetic modifications of floral developmental genes have shown to exert certain function in orchestrating floral organ differentiation as well [2]. These genes involved in flower development are randomly distributed across chromosomes [3]. Disruption of crucial floral developmental genes may cause male or female sterility [4]. In some species, random mutations arose and led to male or female sterility resulting in female or male flowers on different individuals, thereby gradually evolved into dioecy, gynodioecy, androdioecy, and trioecy [3, 4]. The genes causing male or female sterility is regarded as sex-determining genes. Chromosomal rearrangements could result in recombination suppression in a genomic region containing sex-determining genes, leading to the emergence of Sex-determining region (SDR) and the rise of sex chromosome [5, 6]. Despite some progress has been made in the last decade, how distinctive sex-determining genes differentially control flower development remain to be elucidated for most dioecious species.

Trioecious papaya harboring a pair of nascent XY sex chromosomes is an ideal system to study sex differentiation in plants. It has three sex forms. Wild papaya is dioecy and cultivated papaya is gynodioecy. Phenotypic sex reversals can be observed under environmental stress [7, 8]. The combinations of X and Y sex chromosomes determined the sexes of papaya individuals with XX female, XY male, and XY^h^ hermaphrodite. Sex-determining gene(s) have been mapped in 8.1 Mb Sex-determining Region (SDR) in Y or Y^h^ chromosome [9]. Genomic evidence demonstrated that Y^h^ chromosome of hermaphrodite papaya diverged from its Y chromosome ancestor in a wild population about 4000 years ago during the domestication process [10]. Comparative analyses between gynodioecious and dioecious papaya provide an opportunity to study the causative mechanism underlying differential sexual characteristics and the evolutionary changes of sex chromosome systems. In papaya production field, epigenetic sex reversals are often observed when papaya trees are under stresses such as heat or drought. Hermaphrodite flowers abort carpels and reverse to functional male flowers [11]. These floral abnormalities caused by adverse environmental conditions have a negative effect on fruit production, leading to reduced yield [12, 13]. Uncovering the underlying mechanism of sex determination and maintenance process would result methods to improve papaya with no sex reversal to increase fruit production.

Similar to dioecious papaya, the sex of white campion (*Silene latifolia*) and poplars (*Populus* spp.) was determined by XY sex chromosomes. In poplar, sex-specific methylation alterations and sex-biased gene expression were observed in flower and xylem tissues, and thought to play the complex roles in sex expression and sex chromosome evolution [14, 15]. Silencing of the *ARR17* (encoding a phytohormone-associated signal-transduction molecule) through male-specific DNA methylation served as a sex switch in poplar [16]. RNA-directed DNA methylation targeting the intact *ARR* gene contributes to sex determinations in related poplar species [17]. Additionally, male-specific *FERR-R* copy (*ARR17* inverted repeat), a femaleness suppressor of *Populus deltoides*, also generates siRNAs suppressing *FERR* function and guiding differential methylation status of functional female-promoting *FERR* gene (resembling *Arabidopsis thaliana ARR17*) in the sex determining region [18]. In *S. latifolia*, the expression profile changes of sex-linked genes may be associated with DNA methylation and histone covalent modifications of sex chromosomes [19, 20]. Our previous study also showed the distinctive methylation characteristics of dioecious papaya flowers [21]. But it is still not clear what the relation between sex-specific methylation profiles and sex expression is. Do genome-wide methylome differences emerge independently of sex expression, or are they the cause or consequence of sex differentiation in papaya? Are there any distinguishing epigenetic characteristics of nascent XY sex chromosomes? Differentially expressed genes in male and female flower comparison or rudimentary-fertile gynoecium comparison were most overrepresented in phytohormone signal transduction pathways, which may influence sex differentiation and methylome landscapes in papaya [8].

Our aim is to investigate the mechanisms underlying sex-specific methylation landscapes through comparative analyses of both dioecious and gynodioecious papaya grown in spring and summer. Comparing the variances and dynamics of methylome and transcriptome in early-stage of trioecious flowers, we found that genome-wide seasonal changes of sex-specific methylation landscapes was the result of methylome reprogramming partly caused by complex transcriptional regulation of methylation-modification-related and chromatin-remodeling-related genes. Phytohormone signal transduction pathway in male flowers has played an important role in the formation of differential sex characteristics. The study revealed genic and epigenetic regulatory changes in trioecious papaya.

**Figure 1:**
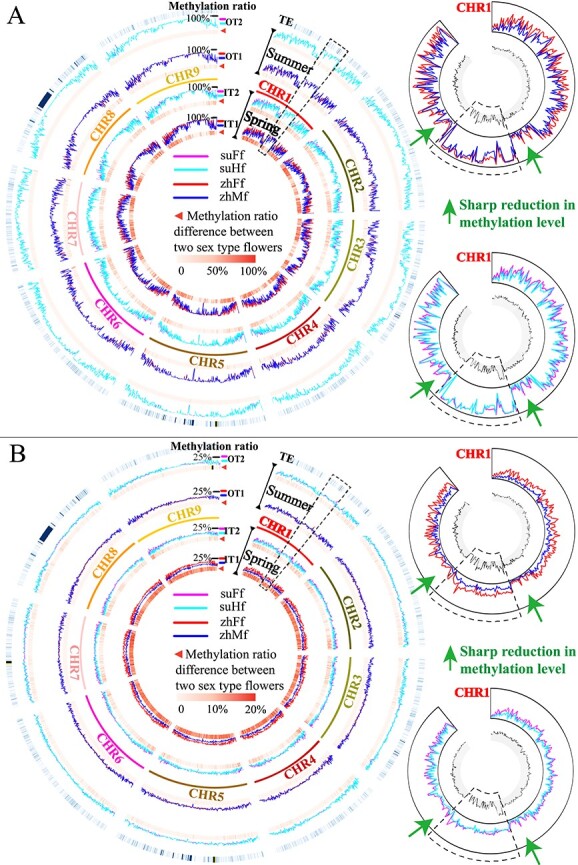
Non-CpG context methylome landscapes of early-stage flowers of the opposite sexes in dioecious and gynodioecious papaya. A&B, CHG and CHH context methylome landscapes of the opposite sexes in dioecious and gynodioecious flowers. Left cyclic graph presented 5 tracks. Of these, outermost track indicates the distribution density of TEs (transposon elements, all sequences were obtained from TE database [22, 23] and mapped to genome), inner two tracks (IT1 and IT2) show the methylation landscapes of opposite sexual flowers grown in spring, and outer two tracks (OT1 and OT2) depict methylome profiles of opposite sexual flowers grown in summer. In these graphs, the methylation ratios of chromosomal segments in dioecious flowers (at 100-Kb resolution) were shown using red curves (zhFf, female flowers of papaya “Zhonghuang”) and blue curves (zhMf, male flowers of papaya “Zhonghuang”) in tracks IT1 and OT1; similarily, methylation ratios of chromosomal segments in gynodioecious flowers were displayed using magenta curves (suFf, female flowers of papaya “SunUp”) and turquoise curves (suHf, hermaphodite flowers of papaya “SunUp”). And the methylation ratio differences between the opposite sexes of dioecious or gynodioecious flowers were displayed using heatmap at bottom of tracks IT1, IT2, OT1 and OT2, which ranged from light red to dark red representing the methylation ratio difference values from the smallest to the largest. The upper-right and lower-right circle-graph particularly shows the methylome profiles of Chromosome 1 (nascent sex chromosome) in dioecious and gynodioecious papaya grown in spring. Fan-shaped frame in dashed lines indicate the SDR counterpart in Chromosome 1. As seen in figure (please magnify the vector graph to show the full detail), the different shape forms of methylome ratio curves between opposite sexual flowers grown in spring means their sex-specific methylome landscape, while the closely overlapped methylome ratio curves in dioecious or gynodioecious flowers grown in summer suggested the very similar methylome landscape due to corresponding methylome reprograming processes. And the methylation ratios of SDR are significantly higher than that of its flanking pseudoautosomal region in Chromosome 1 for all samples.

**Figure 2:**
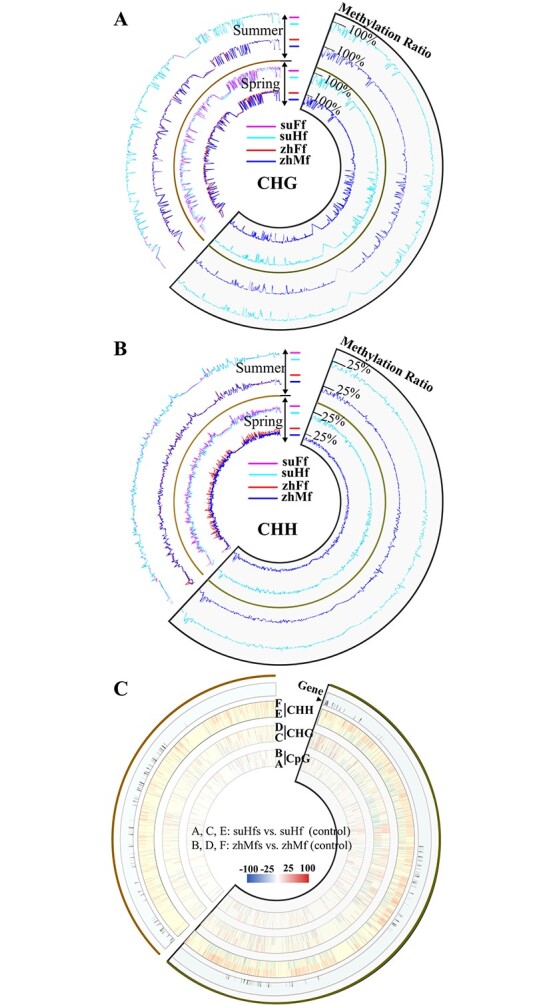
DNA methylation state and variances in non-recombining regions of the nascent sex chromosome. A&B, CHG and CHH context methylation state of the opposite sexes in dioecious and gynodioecious flowers. Each cyclic graph consists of 4 tracks. Of these, inner two tracks show the methylation landscapes of opposite sexual flowers gown in spring, and outer two tracks depicted methylome profiles of opposite sexual flowers gown in summer. In these graphs, the methylation ratios of genomic segments in dioecious papaya “Zhonghuang” were shown using red curves (zhFf, female flowers) or blue curves (zhMf, male flowers); similarily, methylation ratios of genomic segments in gynodioecious papaya “SunUp” were displayed using magenta curves (suFf, female flowers) and turquoise curves (suHf, hermaphodite flowers). Fan-shaped frame in line indicate SDR of Y chromosome. As seen in figure, like chromosome-scale methylome states, the outline of X counterpart methylome ratio curves between opposite sexual flowers gown in spring were different, while the methylome ratio curves of X counterpart were closely overlapped in dioecious or gynodioecious flowers gown in summer. C, methylome variability in SDR and X counterpart of male and hermaphrodite flowers. Cyclic graph consists of four tracks. The distribution of predicted gene was indicated in outermost track, and the significant methylation ratio differences between summer male flowers and spring male flowers (control) in context of CpG, CHG, and CHH were displayed using heatmaps in sub-tracks A, C, and E, which ranged from blue to red representing the difference values from −100% to 100% in specific genomic positions. Similarly, the position-specific significant methylation ratio differences between summer hermaphrodite flowers and spring hermaphrodite flowers (control) were shown in sub-tracks B, D, and F in the same way.

## Results

### Bisulfite sequencing of different sex type flowers library

Bisulfite sequencing of genomic DNA was carried out from early-stage flowers of dioecious and gynodioecious papaya grown in two seasons (summer and spring) in order to investigate seasonal methylomic variances and dynamics among different papaya sex types. The sequencing depth of each library is more than 96×. The quality assessment of bisulfite sequencing data showed that the average calculated methylation ratios of unmethylated control (exogenous λ DNA) in 20 libraries were 0.23% - 0.59%, indicating that the error rate caused by bisulfite conversion failures or sequencing problems was lower than 0.59% and the bisulfite conversion efficiency was therefore obviously higher than 99.41% (Supplementary Table 1). The genomic bisulfite conversion was further evaluated using chloroplast and mitochondria genomes (both organelle genomes were thought to be unmethylated or methylated at extremely low level). The average cytosine methylation ratios of mitochondria genome in all samples were relatively similar, ranging from 0.31% to 0.71%, while that of chloroplast genome in two papaya varieties were quite different, in which average methylation ratios of chloroplast genome were 0.47% - 0.97% in variety “Zhonghuang” (non-transgenic papaya) flowers and 4.36% - 7.98% in variety “SunUp” (transgenic papaya containing coat protein of Papaya Ring Spot Virus through bombardment transformation) flowers, respectively. Unusually high methylation ratios of chloroplast genome in “SunUp” should be false positives due to the alignment error caused by mapping some reads of nuclear plastid sequences (chloroplast DNA-derived sequences inserted in the nuclear genome), which could be methylated, to unmethylated chloroplast genome.

**Figure 3:**
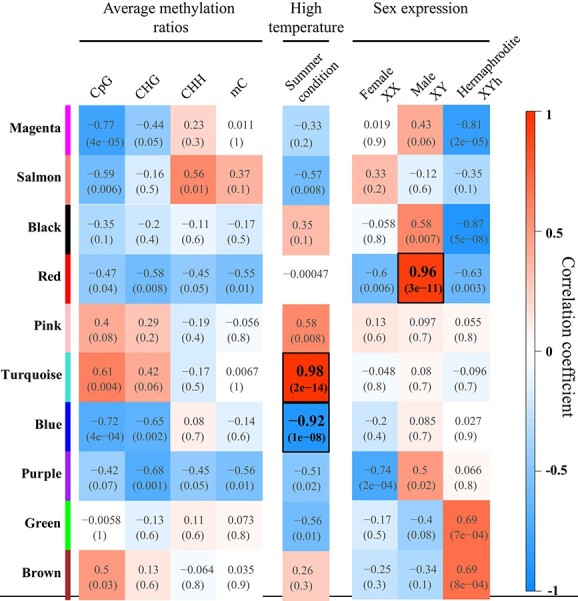
Heatmap of the relevance between the gene module and special traits. Turquoise and blue modules were most closely related modules to summer condition, as well as red module was highly relevant to male sex expression (XY genotype). Correlations between gene modules and CpG, CHG or CHH methylation modification levels were weak (|correlation coefficient| < 0.8).

### Sex-specific methylation landscapes and their seasonal changes

The genome-wide methylation ratios were calculated based on common cytosines that were observed in all samples. CpG, CHG and CHH (H stands for A, T or G) methylation ratios were 80.09–82.88%, 59.03–62.71%, 3.73–9.13% in dioecious flowers, and 82.58–83.73, 62.14–62.89, 5.80–6.99% in gynodioecious flowers (Supplementary Table 2). Comparative analysis of floral samples revealed global methylation characteristics and their changes of CpG, CHG and CHH methylation levels in different seasons. CHG and CHH context methylation patterns show sex-specific and seasonally changing characteristics: (i) The CHG methylation levels between female and male flowers in spring dioecious papaya had significant differences (female 60.59–62.45%; male 59.03–60.50%), while that between female and hermaphrodite flowers in spring gynodioecious papaya were at similar levels (female 62.51–62.89%; hermaphrodite 62.18–62.25%). However, among summer floral samples, we found that average CHG methylation ratios of all samples (containing dioecious and gynodioecious flowers) were at the same levels (approximately 62–63%). (ii) CHH methylation levels of opposite sexes exhibit distinct variations across season. For dioecious flowers, CHH methylation levels of female flowers had declined from 6.05–9.13% (in spring) to 5.71–5.91% (in summer), while that of male counterpart had risen from 3.73–5.45% (in spring) to 6.13–6.49% (in summer). Similarly, a CHH methylation level downtrend in female flowers and an uptrend in hermaphrodite counterpart were observed in summer gynodioecious flowers comparing to spring same-sex flowers. However, there were no significant differences in CpG methylation ratios between opposite sexes of dioecious or gynodioecious flowers collected at the same season.

Further visualizations of CHG and CHH methylome in chromosomes using Circos plot provided a global view of methylome variances and dynamics in papaya flowers in different sex forms and growing seasons (Figure 1). When comparing the chromosome-scale non-CpG context methylation levels of two sex type flowers of dioecious papaya grown in spring by 100 Kb bin chromosome walking, we have found lower average methylation ratios of male flowers (XY genotype) in most chromosomal regions. Similarly, in spring gynodioecious papaya, genome-wide average methylation levels of hermaphrodite flowers (XY^h^ genotype) were marginally below that of female flowers. However, in summer, methylome landscapes of flowers had significantly shifted. We have observed chromosome-scale average methylation levels of early-stage flowers of the opposite sex, in either dioecious or gynodioecious papaya, generally tended to be at the similar level.

**Figure 4:**
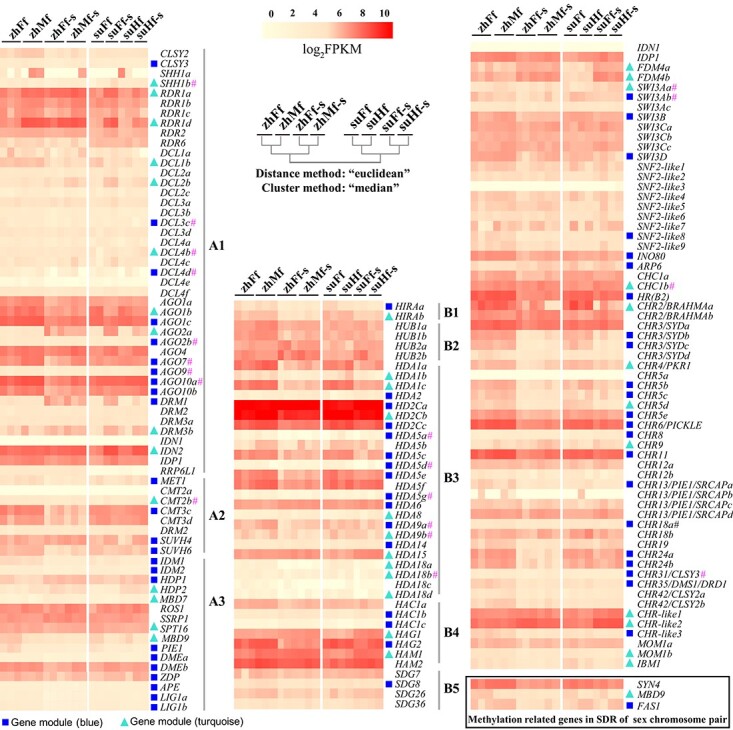
Methylation related gene expression pattern changes in sample groups. A (DNA methylation genes): genes involved in RNA-directed DNA methylation (A1), methylation maintenance (A2) and demethylation (A3). B (histone modification genes): genes similar to Histone Chaperone HIRA (B1), HISTONE MONOUBIQUITINATION1 (B2), histone deacetylase (B3), histone acetyltransferase (B4), SET-domain group protein (B5). C: chromatin remodeling genes. A hierarchical cluster tree of sample groups was created using the “median” method (Weighted center of mass distance algorithm, WPGMC) based on “Euclidean” distance metric. Genes contained in blue and turquoise gene-modules of WGCNA were indicated by blue rectangle and turquoise triangle symbol. Of these genes in modules blue and turquoise, hashes (#) designated the genes didn’t show significant expression difference between summer and spring samples.

We have paid special attention to the SDR counterpart of chromosome 1 (sex chromosome), and found its dramatically higher methylation modification bordered with sharp methylation levels reduced on both sides (proximal pseudo-autosomal region). The results suggested methylation modification in this non-recombining region was heavy, but it did not expand to whole nascent sex chromosomes in papaya.

### DNA methylation in non-recombining regions of the nascent sex chromosome

SDR of the nascent Y chromosome has no recombination with its X counterpart in nascent X chromosome due to two inversions in the SDR of papaya [9]. Based on the reported SDR and X counterpart region assembly sequences, we calculated the methylation ratios and portrayed the methylation landscapes of nonrecombining regions of sex chromosome at 10-kb resolution. The results showed that most part of non-recombining regions of the nascent sex chromosome were heavily methylated up to around 100%, 90% in CpG, CHG contexts. Like chromosome-level exploration results, differential methylation levels of X counterpart between the opposite sexual flowers in dioecious or gynodioecious papaya were observed in non-CpG contexts when comparing spring samples, as well as relatively similar methylation levels were displayed in comparison of summer samples (Figure 2 A, B).

To further explore the stability and variability of stress-responsive epigenome in non-recombining regions of the nascent sex chromosome after hermaphrodite papaya diverged from its male ancestral population, we have investigated the single-base-pair-resolution methylation variances by contrasting summer flowers to corresponding spring ones. The majority of significant stress-responsive methylation changes was not overlapped in chromosomal positions between hermaphrodite and male flowers (Figure 2 C). Particularly, a significant increase in CHH methylation modification widely distributed in SDR of male flowers under the high temperature when comparing spring and summer samples whereas the hermaphrodite flowers had not showed the same trends.

### Integrated analyses reveal critical gene-modules associated with methylation modification and sex expression

Based on global gene expression profiles of floral samples with different sex forms, we employed gene-module-centric co-expression network analyses to investigate the correlations among genomic methylation levels, flower sex expressions and environmental effects. A total of 17 220 genes, consisting of autosomal and sex-chromosomal genes, was selected to construct a gene co-expression network (Gene id and FPKM values of 20 samples were listed in Supplementary Table 3). Genes with similar expression patterns were classified into corresponding gene modules using WGCNA R package. We filtered out 11 gene modules with *p*-values of module-trait relationship at any module-trait pairs more than 0.01, and eventually retained 10 modules for further analysis (Figure 3, Supplementary Table 4). There was not an individual gene module that had a significantly quantitative association to CpG, CHG or CHH genomic methylation levels, considering that their absolute value of the correlation coefficient was less than 0.8. Besides, we found that the red gene module was positively correlated with male (XY genotype) sex expression (with correlation coefficient 0.96) as well as turquoise and blue gene module are closely related to seasonal changes from spring to summer (correlation coefficient 0.98 and − 0.92). The gene expression in red and turquoise, blue gene module was strongly associated with male sex expression and high temperature in summer.

**Figure 5:**
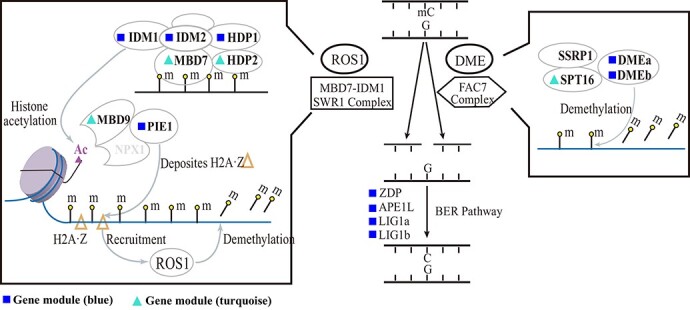
MBD7-IDM/SWR-ROS1 and FACT-DME mediated active DNA demethylation through base excision repair pathway. Blue rectangle and turquoise triangle symbols indicated those genes in module blue and turquoise. These marked genes also showed significantly differently expressed between summer and spring samples. The illustration of active DNA demethylation pathway was modified from previous literature [24].

**Figure 6:**
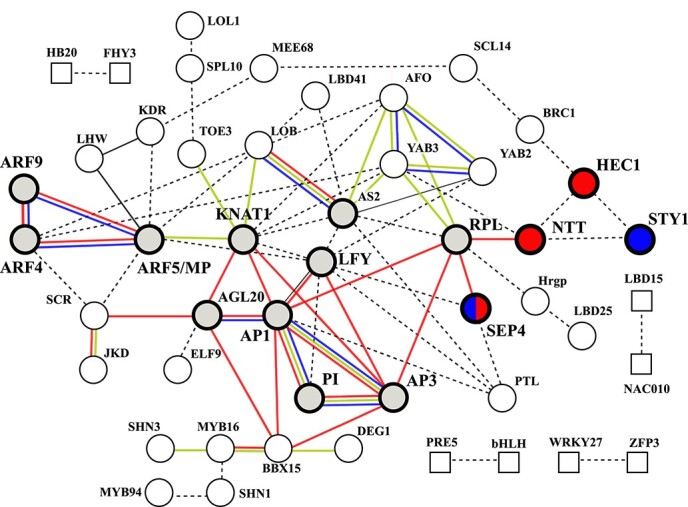
TF interaction analyses predicted a floral development network connection. According to database annotation, SEP4, NTT and HEC1 (in red) were identified to participate in the gynoecium development, as well as SEP4 and STY1 (in blue) involved in the androecium development. STRING interaction scores with the high confidence (0.7). Protein–protein associations of TFs were represented by edges in red (experimentally determined), blue (gene co-occurrence), yellow (co-expression in a species) and dotted line (text mining) according to STRING protein–protein Network Interact Evidence setting. Corresponding TF names and their encoding gene IDs see Supplementary Table 6.

To identify key genes affecting methylation status, we selectively profiled the expression of 170 methylation-related genes (genes involved in DNA methylation, histone modification and chromatin remodeling processes) according to previous reports [21]. Many methylation-related genes were differentially transcriptionally expressed when flowers were growing in different seasons (Figure 4, Supplementary Table 5). Especially in dioecious flowers, majorities of methylation-related genes exhibited a declining expression in summer. For gynodioecious flowers, the gene expression didn’t change extensively when exposed to high temperature in summer. There were 62 methylation-related genes in the blue gene module, as well as 40 genes in the turquoise gene module, which account for 60% of 170 chosen genes. Two methylation-related SDR genes, *MBD9* and *FAS1*, were identified and classified into turquoise and blue gene module, respectively. Thirteen of 16 genes involved in DNA demethylation process in blue and turquoise gene modules had significant differences in their gene expression levels when comparing summer and spring samples (Figure 5). These genes encode crucial proteins in ROS1 (recruited by MBD7-IDM/SWR complex) and DME (recruited by FACT complex) dependent active DNA demethylation pathway through base excision repair (BER).

### Interaction network analysis of transcription factors highlighted auxin-response related floral development in male flowers

WGCNA analysis indicated that the red gene module was tightly associated with male sex expression (XY genotype). We extracted transcription factor genes and analyzed their interaction network in red gene express module to assess the underlying biological plausibility and propose molecular mechanism. Protein–protein interaction network of transcription factors (TF) was constructed using STRING database. TF interaction analyses have established and highlighted a floral development network connection, consisting of reproductive phase transition, floral meristem identity and floral organ developmental factors (Figure 6). Of these floral TFs, four gynoecium and androecium developmental factors (SEPALLATA4/SEP4, NO TRANSMITTING TRACT/NTT, HECATE 1/HEC1 and STYLISH1/STY1) could be connected to ARFs (Auxin Response Factors) though KNAT1(KNOTTED-LIKE FROM *ARABIDOPSIS THALIANA*), RPL(REPLUMLESS) and developmental factor associated with reproductive phase transition.

On the other hand, we performed differential expression analysis by comparing male flowers (grown in spring and summer, 6 samples) against other sexual flowers (14 samples), to identify 778 male-biased genes with significant differential expression. Of these genes, 440 genes were found in WGCNA red gene module which was most strongly related to male floral sex expression (Supplementary Figure 1 and Supplementary Table 6). KEGG-based metabolic pathways enrichment analysis of 440 overlapped genes revealed auxin-responsive signal transduction (especially those in auxin signaling, shown in Supplementary Figure 2), fatty acid degradation pathway, brassinosteroid biosynthesis and glycolysis/gluconeogenesis were overrepresented. Enhanced auxin signaling played a more critical influence in male flower development than that of other flowers. A significantly and steadily transcriptional elevation of *ARFs* (*ARF5* and *ARF9*) exits in male flowers when comparing transcriptomic profiles between male and other sex type flowers across two seasons (Supplementary Table 6).

## Discussion

### Distinctive methylation landscapes may be established and orchestrated by numerous methylation modification regulatory genes and not result from different sex expression

From comparative analyses of whole genome bisulfite sequencing among sex types, we had a new perspective on methylome landscapes of very early-stage flowers in dioecy and domesticated gynodioecy breeding systems. We found differential methylation levels between two opposite sex type flowers grown in spring and then similar methylome profiling in the following summer. Since these significant seasonal changes of methylome landscapes between opposite sexes in both dioecy and gynodioecy systems have arisen along with the gene expression shift of some methylation modification enzymes and chromatin structure regulatory proteins, there must be some connections between them.

Genomic DNA methylation states was susceptible and variable to environmental changes due to adaptive epigenetic reprogramming in many organisms [25, 26]. Specially for plants, genome-wide CHG and CHH sequence contexts DNA methylation modification, regulating by methylation-related genes, displays a high level of plasticity with response to environmental stimuli [27]. We observed that sex-specific methylation landscapes became more similar in opposite sex types after papaya has experienced the seasonal transition from spring to summer. This phenomenon was likely to be caused by the methylome reprogramming mediated through numerous summer-stress-responsive methylation modification and chromatin structure regulatory genes under high temperature stress, such as those methylation related genes contained in blue and turquoise modules which were closely related to summer high temperature condition. Our joint transcriptome analyses further highlighted that the active DNA demethylation likely played a critical role in methylome reprogramming, considering that 13 of 16 genes involved in the known active-demethylation pathway were belong to blue and turquoise modules and differentially expressed at a statistically significant level when flowers are growing from spring to summer. It has been reported that higher methylation frequency was detected when weakening active demethylation effects in several species [28–30]. Genomic DNA demethylation regulation was critical to abiotic stress adaptability [31]. Thus, active demethylation regulation was likely involved in the establishment of a closely similar methylation profile between sex types in summer in response to high temperature.

Furthermore, when analyzing these seasonal methylome variances in different perspectives, such as the relation between sex expression and methylation levels, we speculated that the different sex expression in dioecious or gynodioecious papaya should not be the consequence of distinct chromosome-scale methylation landscapes at the early floral developmental stage. In our study, early-stage flowers of nearly identical development status (without gynoecium and androecium initial differentiation) were used for the sequencing in order to exclude the influences of the developmental differentiation and tissue variability after the appearance of typical sex expression in flowers, thus either different or similar chromosome-scale methylation landscape should be established before the emergence of distinctive sexual characteristics in each individual flower, or independent of sex expression system. When entering to summer, especially in dioecy papaya, with the similar methylome re-established by epigenetic reprogramming, the sexual characteristics could still remain steady, which suggested that there was not the same genetic factor/factors affecting sex-specific methylome variances (at chromosome-level) as controlling the sex expression.

Focusing on sexually polymorphic species, we noticed sex-specific methylation patterns of poplars (*Populus* spp.) were observed and demonstrated in their flowers (*Populus tomentosa*) and xylem (*Populus balsamifera*) tissue [14, 15]. And their comparative transcriptome analysis characterized sex-biased transcriptional expression of two genes related to DNA methylation, *METHYLTRANSFERASE1* and *DECREASED DNA METHYLATION 1* in the sex determination region, which was thought to affect DNA methylation levels between opposite sexual flowers [32]. Similarly, there are two genes (*MBD9* and *FAS1*) related to DNA methylation locating in the sex determination region of papaya. More recently, MBD9 was found to be interacted with chromatin remodeling proteins to assemble SWR1 complex, and then deposited H2A.Z to facilitate ROS1-mediated demethylation [33]. FAS1 was reported to participate in auxin-TIR1/AFBs signaling responsive chromatin accessibility pathway [34], and may affect methylation modification status in enhanced-auxin-responsive male flowers. Thus these two genes (*MBD9* and *FAS1*) with differential expression may also involve in distinctive methylation landscapes shaping, since the plants of opposite sexes from either dioecious or gynodioecious papaya have identical genetic background in vast majority of the genome except 8.1 Mbp of the SDR and 3.5 Mbp of the X counterpart, representing the only difference of genomic sequence in individuals with opposite sexes.

### Non-recombining region methylation patterns revealed the epigenetic characteristics of nascent XY sex chromosomes

Sex chromosomes evolved from autosomes [35]. The existence of SDR is the distinguishing features of nascent sex chromosomes in land plants [3, 6]. We dissect DNA methylation landscapes of all samples and observed that hypermethylated SDR as well as its relatively hypomethylated pseudo-autosomal flanking segment particularly outlined methylation signatures of primitive XY sex chromosomes in papaya, which emerges in close proximity to the centromere and now contains the centromere [36, 37]. We speculated the barrier effects distinguishing the centromeric SDR from flanking pseudo-autosome may emerge after the SDR formation, considering that centromeric- and paracentromeric chromosomal segment of other chromosomes did not show similar epigenetic characteristics. Earlier cytological findings indicated SDR of nascent Y chromosomes were likely initiated near the hypermethylated and heterochromatinized centromere in two *Caricaceae* species (*Carica papaya* and dioecious *Vasconcellea parviflora*) [36, 38]. In our study, we observed the SDR of sex chromosome was hypermethylated. If some unknown mechanisms prevent centromeric hypermethylation expand to closely flanking pseudo-autosomal region in sex chromosomes, the repetitive DNA insertion over-accumulation in SDR would further expand the SDR with heavy methylation modification. The hypermethylated SDR would be easily heterochromatinized compared to relatively hypomethylated pseudo-autosome and might affect sex chromosome behavior and evolution.

Although Y^h^ chromosome had evolved from Y chromosome for 4000 years, there seems no great changes between them in terms of the chromosomal methylation characteristics, which suggested that the sex divergence did not greatly affect the chromosomal methylation landscapes.

### Differential gene expression in male flowers unravelled possible auxin-response driven sex determination mechanism and epigenetic influence

In previous study, we have identified the enhanced auxin response and upregulated *ARFs* (*ARF4* and *MP/ARF5*) transcription in male flowers comparing to female or hermaphrodite flowers [8, 21]. Based on interaction network analyses, we proposed that ARFs (ARF4 and MP/ARF5) served important functions and interacted with KNAT1 and RPL to trigger the floral developmental cascade genes in male-associated gene module, consisting of the floral meristem identity and floral development genes. Of these, some associated cascade genes were proved to be critical for floral sexual structure development in other species. In *Arabidopsis*, as the floral development continues, *KNAT* activated the expression of *RPL*, then two transcription factors combinations (RPL-NTT and NTT-HEC1) promote the formation of the female reproductive structures in flowers [39, 40]. In our study, *AP3, PI, RPL, NTT* and *HEC1* were identified in the specific male-associated gene module and could be integrate into a developmental cascade. Male-biased expression of ARFs links with these crucial developmental factors to potentially trigger floral developmental cascade, leading to differentiated sexual characteristics. It was reported that some differentially expressed *ARFs* were identified between perfect hermaphrodite flowers and summer female sterile flowers (with the sex reversal from hermaphrodite to male flowers) in hermaphrodite papaya and *ARFs* can coordinate the development of floral organs at flower maturation stages [41, 42]. Our findings on ARFs-mediated regulatory network of early-stage flowers would fill in the critical knowledge gap of floral specification and patterning in papaya. The diverse functions of *ARFs* members at consecutive floral developmental stages will be validated in future projects. For producers, utilizing the plant growth regulators that affect the auxin homeostasis may reduce the occurrence of floral abnormalities and deformed fruits.

Another interesting finding was that differentially expressed genes were over-represented in fatty acid degradation and auxin-responsive signal transduction in comparison between male flowers and flowers with different sex types. It was previously inferred that fatty acid and auxin signaling can influence chromatin conformational adjustment [34, 43]. DNA hypermethylation at several genomic loci, along with reduced histone acetylation modification, resulted from attenuating fatty-acid beta-oxidation degradation in Arabidopsis [43]. TIR1/AFBs auxin signaling pathway could decrease chromatin accessibility in proliferative tobacco cells [34]. The differences of fatty acid degradation and auxin-responsive signal transduction pathway may also have an influence in the methylation and chromatin modification of male flowers, which reflect the complexity of epigenetic changes after sex differentiation in papaya.

Our findings suggest that DNA methylation is involved in the sexual differentiation and sex chromosome evolution. Distinct phytohormone signaling process and differential methylation-related gene expression, which often occur in sexual plants, contributed to the DNA methylation alteration in papaya. Similar phenomenon was observed in the garden asparagus (*Asparagus officinalis*) [44], which reinforced our conclusions.

## Materials and methods

### Plant materials

Two-year-old gynodioecious papaya variety “SunUp” and dioecious papaya variety “Zhonghuang” were planted in the greenhouse of Fujian Agriculture and Forestry University (Fuzhou, P. R. China). Gynodioecious “SunUp” is ring spot virus (PRSV) resistant transgenic papaya cultivar [45, 46], and dioecious “Zhonghuang” is an improved papaya variety with full-sib mating for several generations [8, 21] (Supplementary Figure 3).

The humid tropical climate in Fuzhou region is suitable for papaya growth. All plants were transplanted in pots (1 m height and 1 m diameter) with garden loam, and watered by microsprinkler irrigation system twice a week to avoid drought stress. We collected early-stage flowers (flower length < 1.5 mm, with no differentiated structure of gynoecium and androecium) for the DNA and RNA extraction. There were three male- or female flower biological replicates from three male or female papaya var. “Zhonghuang” individuals respectively (one single biological replicate was collected from a tree individual), whereas two female- or hermaphrodite-flower replicates from four female or hermaphrodite “SunUp” individuals (one single biological replicate was taken from two tree individuals with the same sex form). Because hermaphrodite and female flowers in gynodioecious papaya are scarce, making it difficult to obtain adequate sample amounts from a single tree individual.

The spring flowers (collected in April 2017, 24°C) and summer flowers (collected in August 2017, 39°C) were used in this study. Ambient temperatures at 39°C are harsh for papaya growth in the greenhouse, which were thought to be adverse stress conditions.

Female and male flowers of papaya var. “Zhonghuang” as well as female and hermaphrodite flowers of papaya var. “SunUp” grown in the spring were respectively designated as zhFf, zhMf, suFf and suHf. Similarly, when flowers were collected in summer, zhFfs and zhMfs were refer to female and male flowers of papaya “Zhonghuang”, while suFfs and suHfs represented female and hermaphrodite flowers of papaya “SunUp”. Multiplex RT-PCR detected and exclude virus infection of study samples [47].

### Whole genome bisulfite sequencing and transcriptome sequencing

Each sample was divided into half for both the DNA methylation sequencing and transcriptome sequencing. A modified method of bisulfite-sequencing library construction was adopted for low initial amount of total DNA (200 ng genomic DNA mixing with trace amounts of exogenous unmethylated λ DNA). RNA-seq libraries were prepared with Ultra RNA Library Prep Kit (NEB, #E7770L). 100-nt or 150-nt pair-end reads were generated by high throughout sequencing, all of the indexed libraries using Illumina HiSeq2500 platform.

### DNA methylation pattern and variance analysis

We retrospectively analyzed the methylation profiling of dioecious flowers from papaya “Zhonghuang” and allocated gynodioecious flowers for further conjoint analyses following previous reported procedure. In brief, we aligned clean reads to papaya new reference genome (Cpapaya_SunUp.v5, chromosome-scale de novo genome assembly with PacBio long reads, chromosome 1 is defined as sex chromosome) and its organelle genome as well as exogenous unmethylated λ DNA control by Bismark pipeline [48] and then processed to obtain the methylation ratio/frequency (methylated cytosines/total observed cytosines at a given locus) with single base-pair or 100Kb bin resolution using “methykit” R package program [49]. Besides, in this process, the bisulfite conversion rate of each library was evaluated from the average methylation ratios of exogenous unmethylated λ DNA (Enterobacteria phage lambda genome, Genbank accession J02459.1) and endogenous organelle DNA (papaya chloroplast genome, Genbank accession EU431223.1; papaya mitochondrion genome, Genbank accession EU431224.1). The differential methylation cytosines (DMCs) calling with false positive errors filtering was done using the same method as our earlier report presented [21]. The cytosine loci with a minimum methylation ratio difference at 25% (q-values <0.01) were considered significant DMCs.

### Gene co-expression net analysis and differentially expressed gene (DEG) analysis

The same samples were also used for RNA-seq analysis. We extracted total RNA with Tripure reagent (Roche, Cat.No.11667165001) and constructed RNA-seq libraries with Ultra RNA Library Prep Kit (NEB, #E7770L). Illumina HiSeq2500 system was used to perform the 100-nt or 150-nt pair-end reads high-throughput sequencing.

We aligned clean reads to the published papaya reference genome using STAR aligner and calculated the FPKM (Reads Per Kilobase per Million mapped reads) value of each gene by processing the result bam files with cufflink pipeline [50, 51]. After filtering out of genes with the lower expression (the average RPKM value of 20 samples is less than 0.6), totally 17 221 genes were chosen for downstream weighted gene co-expression and genes-traits association analysis using “WGCNA” R package program [52] (all of FPKM values are added with 0.1 to avoid the problems of statistical analysis in some procedure. β = 12 was selected as the smallest value that is referred to as approximately scale-free topology, and other parameter manipulation and command flow followed the“WGCNA” R Tutorial https://horvath.genetics.ucla.edu/html/CoexpressionNetwork/Rpackages/WGCNA/Tutorials/). In this process, genes with similar expression levels were initially clustered into gene modules. Then we conserved the concerned traits with the corresponding ambient conditions into seven variables (three numerical variables and four binary variables) and analyzed the relevance between the gene modules and concerned traits. Three numerical variables were the CpG, CHG and CHH context genomic average methylation ratios, which reflected the genomic DNA methylated modification levels. Four binary variables were female sex expression (1’s for female flowers with XX sex chromosome genotype, 0’s for others), male sex expression (1’s for male flowers with XY genotype, 0’s for others), hermaphrodite sex expression (1’s for hermaphrodite flowers with XY^h^ genotype, 0’s for others) and high-temperature summer condition (1’s for flowers collected in summer, 0’s for others). Pearson’s correlations between each cluster’s eigengene (gene module) and concerned traits were calculated to explore which gene modules were related to traits.

“Limma”R package program was performed to screen differentially expressed genes between two types of samples. DEGs with adj.P.Val (Benjamini-Hochberg false discovery rate adjusted p-value) < 0.05 were considered as being significant. KEGG pathway enrichment analyses were conducted using KOBAS 3.0 on-line server [25, 26], which background parameters, statistical method and FDR correction were set as previously described.

## Acknowledgments

This work was funded by the grant 2020J011360 and foreign cooperation project 2021I0009 from the Department of Science and Technology of Fujian Province, US National Science Foundation (NSF) Plant Genome Research Program Award (1546890), and National Natural Science Foundation of China (31701889). It was also supported by the science and technology innovation fund (CXZX2020091A) to J. Y. and the startup fund to R.M. from Fujian Agriculture and Forestry University.

## Author contributions

P.Z. and R.M. designed the project. P.Z. performed the experiments. P.Z., X.M., X. Z. analyzed and plotted the HTS datas. J. Y. completed and provided the chromosome-assembly of papaya reference genome, Z. L. checked and improved the analysis results. P.Z., X. Z. and X.M. wrote the manuscript. X. Z., X.M., and R.M. revised the manuscript.

## Data availability

All high-throughput methylome and transcriptome data were publicly accessible in Genome Sequence Archive (GSA, https://bigd.big.ac.cn/gsa) of BIG Data Center (Beijing Institute of Genomics, Chinese Academy of Sciences) under GSA accession numbers CRA002034, CRA001995.

## Conflict of interest

The authors declare that they have no conflict of interest.

## Supplementary Material

Web_Material_uhab065
