# Supplementary material for: Methylation related genes affect sex differentiation in dioecious and gynodioecious papaya
Source: Hortic Res. 2022 Jan 20;9:uhab065. doi: 10.1093/hr/uhab065 (PMC8935930; doi:10.1093/hr/uhab065)
Supplement: Web_Material_uhab065 [file web_material_uhab065.zip › Supplementary_Figure 1.docx]

**Supplementary Figure 1** 440 overlapped genes between red gene module and male-specific DEGs
