# Supplementary material for: Methylation related genes affect sex differentiation in dioecious and gynodioecious papaya
Source: Hortic Res. 2022 Jan 20;9:uhab065. doi: 10.1093/hr/uhab065 (PMC8935930; doi:10.1093/hr/uhab065)
Supplement: Web_Material_uhab065 [file web_material_uhab065.zip › Supplementary_Figure 2.docx]

**Supplementary Figure 2** KEGG analysis of 440 overlapped genes emphasized the phytohormone signaling transduction in male flowers compared to other sexual flowers, red or cyan boxes show increase or decrease at gene transcriptional levels, Ub in yellow circles indicate protein degradation by the ubiquitin-proteasome complex, ①TIR1/AFB complex regulate and trigger the degradation of Aux/IAA by ubiquitination modification, ②Aux/IAA degradations abolish the suppressive effects of transcriptionally expression of ARFs.

AUX1: evm.TU.supercontig_1125.1, TIR: evm.TU.supercontig_27.130, ARFs: ARF5/MP(evm.TU.supercontig_26.24) and ARF9(evm.TU.supercontig_15.1), IAA16: IAA16a(evm.TU.supercontig_10.173) and IAA16b(evm.TU.supercontig_23.159), DFL2/GH3-10: evm.TU.supercontig_34.122, ATRR4: evm.TU.supercontig_64.99, ARR5: evm.TU.supercontig_35.27, SnRK2: evm.TU.supercontig_55.80, TGA: evm.TU.supercontig_32.14.
