# Supplementary material for: Methylation related genes affect sex differentiation in dioecious and gynodioecious papaya
Source: Hortic Res. 2022 Jan 20;9:uhab065. doi: 10.1093/hr/uhab065 (PMC8935930; doi:10.1093/hr/uhab065)
Supplement: Web_Material_uhab065 [file web_material_uhab065.zip › Supplementary_Table 1.docx]

Supplementary Table 1 Summary of bisulfite sequencing data

|  | Sample source | Sample names | Total number of reads pair | Theoretical sequencing depth | Methylation ratios of lambda genome % | Methylation ratios of chloroplast genome % | Methylation ratios of mitochondrion genome % |
| --- | --- | --- | --- | --- | --- | --- | --- |
| Dioecious papaya | Female flowers | zhFf1 | 181774064 | 155 × | 0.234 | 0.609 | 0.339 |
| variety ‘Zhonghuang’ | (spring) | zhFf2 | 317916206 | 272 × | 0.521 | 0.972 | 0.620 |
|  |  | zhFf3 | 311296521 | 226 × | 0.589 | 0.953 | 0.709 |
|  | Male flowers | zhMf1 | 151320125 | 129 × | 0.404 | 0.478 | 0.401 |
|  | (spring) | zhMf2 | 136915437 | 117 × | 0.378 | 0.438 | 0.373 |
|  |  | zhMf3 | 147989616 | 140 × | 0.377 | 0.494 | 0.400 |
|  | Female flowers | zhFfs1 | 112045989 | 96 × | 0.420 | 0.477 | 0.394 |
|  | (summer) | zhFfs1 | 115728174 | 99 × | 0.407 | 0.472 | 0.385 |
|  |  | zhFfs3 | 128433938 | 110 × | 0.402 | 0.549 | 0.394 |
|  | Male flowers | zhMfs1 | 117559902 | 100 × | 0.392 | 0.482 | 0.375 |
|  | (summer) | zhMfs2 | 138246727 | 118 × | 0.406 | 0.509 | 0.391 |
|  |  | zhMfs3 | 135706771 | 116 × | 0.375 | 0.460 | 0.364 |
| Gynodioecious papaya  variety ‘SunUp’ | Female flowers | suFf1 | 132537436 | 113 × | 0.405 | 7.976 | 0.379 |
|  | (spring) | suFf2 | 153850530 | 146 × | 0.509 | 7.632 | 0.486 |
|  | Hermaphrodite flowers | suHf1 | 224692748 | 125 × | 0.442 | 4.364 | 0.440 |
|  | (spring) | suHf2 | 137399431 | 116 × | 0.431 | 7.754 | 0.464 |
|  | Female flowers | suFfs1 | 139883635 | 118 × | 0.479 | 6.096 | 0.449 |
|  | (summer) | suFfs2 | 136663112 | 115 × | 0.392 | 5.288 | 0.369 |
|  | Hermaphrodite flowers | suHfs1 | 137011235 | 116 × | 0.367 | 7.746 | 0.345 |
|  | (summer) | suHfs2 | 138730412 | 117 × | 0.342 | 6.250 | 0.319 |
