# Supplementary material for: Methylation related genes affect sex differentiation in dioecious and gynodioecious papaya
Source: Hortic Res. 2022 Jan 20;9:uhab065. doi: 10.1093/hr/uhab065 (PMC8935930; doi:10.1093/hr/uhab065)
Supplement: Web_Material_uhab065 [file web_material_uhab065.zip › Supplementary_Table 2.docx]

Supplementary Table 2 The average methylation ratios of flower samples (%)

| Sequence contexts  Sample | | CpG | CHG | CHH | mCs |
| --- | --- | --- | --- | --- | --- |
| Dioecious papaya | zhFf1（Female Spring） | 80.09 | 60.59 | 6.05 | 23.17 |
|  | zhFf2（Female Spring） | 81.34 | 62.45 | 8.74 | 25.57 |
|  | zhFf3（Female Spring） | 80.89 | 62.17 | 9.13 | 25.76 |
|  | zhMf1（Male Spring） | 81.21 | 60.50 | 5.45 | 22.88 |
|  | zhMf2（Male Spring） | 80.19 | 59.03 | 3.73 | 21.27 |
|  | zhMf3（Male Spring） | 80.74 | 60.00 | 4.79 | 22.25 |
|  | zhFfs1（Female Summer） | 82.88 | 62.56 | 5.72 | 23.57 |
|  | zhFfs2（Female Summer） | 82.83 | 62.56 | 5.71 | 23.61 |
|  | zhFfs3（Female Summer） | 82.87 | 62.71 | 5.91 | 23.74 |
|  | zhMfs1（Male Summer） | 82.70 | 62.54 | 6.13 | 23.85 |
|  | zhMfs2（Male Summer） | 82.58 | 62.59 | 6.49 | 24.11 |
|  | zhMfs3（Male Summer） | 82.62 | 62.55 | 6.25 | 23.93 |
| Gynodioecious papaya | suFf1（Female Spring） | 82.68 | 62.89 | 6.99 | 24.53 |
|  | suFf2（Female Spring） | 82.97 | 62.51 | 6.47 | 24.12 |
|  | suHf1（Hermaphrodite Spring） | 82.61 | 62.25 | 5.92 | 23.64 |
|  | suHf2（Hermaphrodite Spring） | 82.58 | 62.18 | 6.27 | 24.04 |
|  | suFfs1（Female Spring） | 83.67 | 62.73 | 5.80 | 23.76 |
|  | suFfs2（Female Spring） | 83.66 | 62.70 | 5.81 | 23.75 |
|  | suHfs1（Hermaphrodite Summer） | 83.73 | 62.39 | 6.64 | 24.31 |
|  | suHfs2（Hermaphrodite Summer） | 83.58 | 62.14 | 6.49 | 23.99 |
